# Spiking Neural Networks and Their Applications: A Review

**DOI:** 10.3390/brainsci12070863

**Published:** 2022-06-30

**Authors:** Kashu Yamazaki, Viet-Khoa Vo-Ho, Darshan Bulsara, Ngan Le

**Affiliations:** 1 Department of Computer Science and Computer Engineering, University of Arkansas, Fayetteville, AR 72701, USA; kyamazak@uark.edu (K.Y.); khoavoho@uark.edu (V.-K.V.-H.); 2 Department of Electrical and Computer Engineering, University of California, San Diego, CA 92093, USA; dbulsara@ucsd.edu

**Keywords:** spiking neural networks, biological neural network, autonomous robot, robotics, computer vision, neuromorphic hardware, toolkits, survey, review

## Abstract

The past decade has witnessed the great success of deep neural networks in various domains. However, deep neural networks are very resource-intensive in terms of energy consumption, data requirements, and high computational costs. With the recent increasing need for the autonomy of machines in the real world, e.g., self-driving vehicles, drones, and collaborative robots, exploitation of deep neural networks in those applications has been actively investigated. In those applications, energy and computational efficiencies are especially important because of the need for real-time responses and the limited energy supply. A promising solution to these previously infeasible applications has recently been given by biologically plausible spiking neural networks. Spiking neural networks aim to bridge the gap between neuroscience and machine learning, using biologically realistic models of neurons to carry out the computation. Due to their functional similarity to the biological neural network, spiking neural networks can embrace the sparsity found in biology and are highly compatible with temporal code. Our contributions in this work are: (i) we give a comprehensive review of theories of biological neurons; (ii) we present various existing spike-based neuron models, which have been studied in neuroscience; (iii) we detail synapse models; (iv) we provide a review of artificial neural networks; (v) we provide detailed guidance on how to train spike-based neuron models; (vi) we revise available spike-based neuron frameworks that have been developed to support implementing spiking neural networks; (vii) finally, we cover existing spiking neural network applications in computer vision and robotics domains. The paper concludes with discussions of future perspectives.

## 1. Introduction

The last decade has witnessed the growing abilities of artificial neural networks (ANNs) from the first generation multi-layer perceptron (MLP) to the many state-of-the-art techniques in the second generation deep neural networks (DNNs). This achievement strongly depends on a large amount of annotated data and the widespread availability of high-performance computing devices as well as the general-purpose Graphics Processing Units (GPUs). Despite this great advancement, ANNs still lag behind the biological neural networks in terms of energy efficiency and abilities for online learning. Many attempts have been made to reduce the power consumption of traditional deep learning models. In order to find more compact networks that can achieve similar performance with much less complexity and a smaller number of parameters compared to the original network, many techniques have been developed such as quantization [1], pruning [2], and knowledge distillation [3]. Quantization converts the weights and inputs of the network into integer types, which makes the overall operations lighter than the floating-point operations. In pruning, the connections of a network are iteratively removed during or after the training. To compress a neural network without dropping performance, knowledge distillation transfers complex knowledge learned by a heavy network called a teacher to a lightweight network called a student.

Although ANNs/DNNs are historically brain-inspired, they are fundamentally different in structure, neural computations, and learning rules compared to the biological neural network. This observation leads to the spiking neural networks (SNNs), which are often referred to as the third generation of neural networks that could be a breakthrough of bottlenecks of ANNs. Using SNNs on neuromorphic hardware, such as TrueNorth [4], Loihi [5], SpiNNaker [6], NeuroGrid [7], etc., is worth mentioning and a promising approach to the energy consumption problem. In SNNs, such as in biological neural networks, neurons communicate with each other with discrete electrical signals called spikes and work in continuous time.

Due to their functional similarity to the biological neural networks, SNNs can embrace the sparsity found in biology and are highly compatible with temporal code [8]. Although SNNs still lag behind DNNs in terms of their performance, the gap is vanishing on some tasks, while SNNs typically require much lower energy for the operation. However, SNNs are still difficult to train in general, mainly owing to their complex dynamics of neurons and the non-differentiable nature of spike operations. A comparison between biological neural networks, ANNs, and SNNs is given in Table 1. BP is backpropagation.

## 2. Biological Neurons

Neurons are the basic working units of the nervous system that process information by propagating electrochemical signals through action potentials. Neurons are not electrically neutral nor extracellular fluid because of the presence of ions within them. Ions are constantly moving in and out of the cell through a membrane that can dynamically modify its electric permeability with external electrochemical signals. The flux of ions entering and exiting the cell causes a virtual current flow through the membrane, mostly ascribed to Na${}^{+}$, K${}^{+}$, and Cl${}^{-}$ ions.

**Figure 1:**
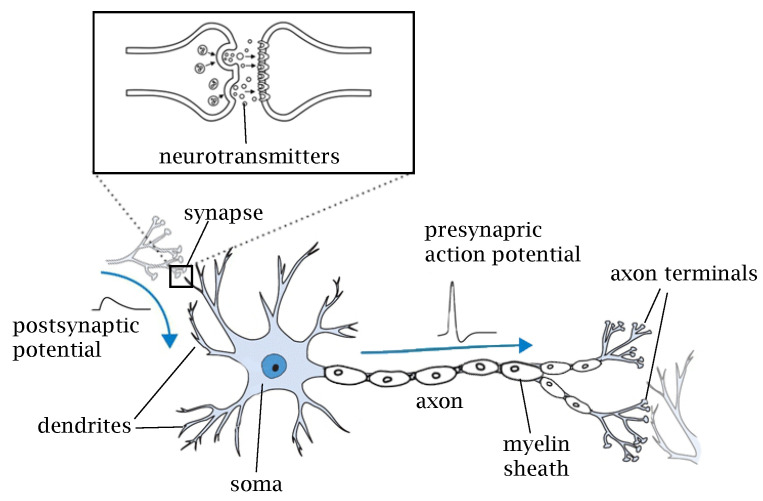
A typical structure of a biological neuron and synapse.

Figure 1 shows a typical structure of a neuron with four main components: dendrites, soma, axon, and synapse. *Dendrites* are the short nervous termination that can be considered as the input of the neuron. They translate the chemical signals carried by neurotransmitters released from the pre-synaptic neuron into electric signals. *Soma* is the cell body where membrane potentials propagated from synaptic inputs are integrated, which ultimately determines whether the post-synaptic cell fires action potentials before being transmitted to the axon. This interaction of influences is called neural integration. *Axon* carries the action potential towards other nerve cells. In order to rapidly carry the action potential at long distances without attenuation, some axons are coated with a myelin sheath. *Synapses* are the contact structure for information transfer that interconnect neurons in a neural network. Synapses can be broadly divided into chemical and electrical synapses. In *chemical synapses*, there is no direct contact between the pre- and post-synaptic neurons. The signal from the pre-synaptic neuron is transmitted via *neurotransmitters* contained in the synaptic granules released into the synaptic cleft. Neurotransmitters bind to receptors in the post-synaptic cell, directly altering membrane potential or activating intracellular secondary messengers to transmit the information. This type of transmission is slow but amplifies the signal and can make the effects of the incoming spike last longer. Chemical synapses can be subdivided into excitatory and inhibitory synapses. *Excitatory synapses* are synaptic connections that depolarize post-synaptic cells through synaptic transmission and promote the firing of action potentials. *Inhibitory synapses* are synaptic connections that hyperpolarize post-synaptic cells by synaptic transmission and inhibit the development of action potentials. Glutamate and GABA are the most common excitatory and inhibitory neurotransmitters, respectively; ionotropic receptors for glutamate are AMPA and NMDA and that of GABA are $GABA_{A}$ and $GABA_{B}$ [9]. *Electrical synapses*, on the other hand, are structures that transmit membrane potential charges directly to the next neuron via gap junctions on the contact membrane. This kind of communication is very rapid since there are no chemical reactions within the transmission; however, there is no gain in signal amplitude as in the chemical synapses.

### 2.1. Membrane Potential

The electric potential inside a cell with respect to the outside of the cell is called the membrane potential. The membrane potential can be derived using the Goldman–Hodgkin–Katz equation, which takes into consideration the relative permeability of the plasma membrane to each ion in question.

(1) $$v_{m}=\frac{RT}{F}\ln\frac{P_{K}{\left[K^{+}\right]}_{out}+P_{Na}{\left[Na^{+}\right]}_{out}+P_{Cl}{\left[Cl^{-}\right]}_{in}}{P_{K}{\left[K^{+}\right]}_{in}+P_{Na}{\left[Na^{+}\right]}_{in}+P_{Cl}{\left[Cl^{-}\right]}_{out}}$$

where *R* is the universal gas constant, *T* is the absolute temperature 310.15 (K) at human body temperature (37 [°C]), *F* is the Faraday constant (=96,485 (C· mol${}^{-1}$)), ${\left(A\right)}_{out}$ is the extracellular concentration of ion *A*, and ${\left(A\right)}_{in}$ is the intracellular concentration of ion *A*, and $P_{A}$ is the membrane permeability for ion *A*, and for a typical neuron at rest, it is known that $P_{K}$:$P_{Na}$:$P_{Cl}=1$:0.04:0.45. In contrast, approximate relative permeability at the peak of a typical neuronal action potential are $P_{K}$:$P_{Na}$:$P_{Cl}=1$:12:0.45 [10].

#### 2.1.1. Resting Membrane Potential

Due to the action of a number of proteins, ions are constantly moving in and out of the cell. Although the influx of ions does not stop, charge transfer becomes apparently immobile when the total charge of the outflowing ions and the total charge of the inflowing ions per unit time becomes the same. The resting membrane potential of a cell is determined by the net flow of ions through the “leak” channels that are open in the resting state. Based on the relative membrane permeability for a typical neuron at rest, we can calculate the resting membrane potential $E_{m}$ as follows:

(2) $$\begin{array}{cc} E_{m} & =\frac{RT}{F}\ln\frac{5.5P_{K}+135\times 0.04P_{K}+9\times 0.45P_{K}}{150P_{K}+15\times 0.04P_{K}+125\times 0.45P_{K}}\\ \; & =-70.15\;[mV] \end{array}$$

Since the reversal potential for Cl${}^{-}$ ion is typically close to the resting membrane potential, the Cl${}^{-}$ ion is usually ignored when discussing a neuron’s resting membrane potential.

#### 2.1.2. Action Potential

When an action potential occurs, sodium channels on the axon are opened and Na${}^{+}$ ions are free to move in and out of the cell membrane. The membrane potential fluctuates accordingly toward the reversal potential of the Na${}^{+}$ ion. The sodium channel is then inactivated and closed, and now the potassium channel, which is potential-dependent, is opened. Now, the membrane potential descends back toward the reversal potential of the K${}^{+}$ ion and undershoots beyond the resting membrane potential $E_{m}$.

(3) $$\begin{array}{cc} v_{peak} & =\frac{RT}{F}\ln\frac{5.5P_{K}+135\times 12P_{K}+9\times 0.45P_{K}}{150P_{K}+15\times 12P_{K}+125\times 0.45P_{K}0}\\ \; & =38.43\;[mV] \end{array}$$

## 3. ANN Models

A rate-based neuron models the activity of a neuron only by the macroscopic feature, firing rate *r*, regardless of the change in membrane potential or spike timing. The first rate-coded artificial neuron, which is known as formal neuron or threshold logic unit, was proposed by [11]. Based on the formal neuron, reference [12] introduced perceptron, using the Heaviside step function as the activation function. These first-generation neurons fire binary signals when the sum of incoming signals reaches a threshold of a neuron. This concept is later extended to utilize continuous activation functions, including the sigmoid [13] or hyperbolic tangent function, to deal with analog inputs and outputs; consequently, this enabled the training of the neural network through a powerful backpropagation algorithm that exploits gradient-descent. Because of the proven ability of a sufficiently large neural network of artificial neurons to approximate any analog function arbitrarily well (universal approximation theorem states that a feed-forward network with a single hidden layer with a finite number of neurons can approximate continuous functions, under assumptions on the non-polynomial activation function [14,15]; Sigmoidal activation function and the ReLU are also proved to follow the universal approximation theorem [16]), artificial neural networks have been widely used as a powerful information-processing tool in machine learning. In general, the discrete-time firing rate model can be formulated as $r=\sigma (\sum_{i}w_{i,j}x_{j})$ and usually grouped together for computational efficiency:

(4) r=f(Wu+b)

where $u\in R^{N_{pre}}$ is the firing rate of pre-synaptic neurons, $r\in R^{N_{post}}$ is the firing rate of post-synaptic neurons, $W\in R^{N_{post}\times N_{pre}}$ is the weight matrix that represents the synaptic strength between the pre- and post-synaptic neurons, $b\in R^{N_{post}}$ is the bias term, and f(·) is the non-linear activation function.

Nowadays, Rectified Linear Unit (ReLU) [17] and its variants are commonly employed as the non-linearity because they tend to show better convergence performance than the sigmoidal activation function [18]. This formulation of the group of rate-based neurons is often referred to as a fully-connected layer. The modern architecture of neural networks stacks a variant of this layer to create very deep networks of neurons, which is often referred to as deep neural networks (DNNs). Neural networks are typically called deep when they have at least two hidden layers computing non-linear transformations of the input. One of the commonly used building blocks of DNNs is a convolutional layer. A convolutional layer is a special case of the fully connected layer that implements weight sharing for processing data that has a known grid-like topology, e.g., images. Because of this inductive bias, convolutional neural networks (CNNs) [19,20] can utilize the spatial correlation of the signal in a more sensible way. The representational properties of early layers in the CNNs are similar to the response properties of neurons in the primary visual cortex (V1), which is the first cortical area in the visual hierarchy of the primate’s brain. CNNs possess two key properties that make them extremely useful for image applications: spatially shared weights and spatial pooling. This kind of network learns features that are shift-invariant, i.e., filters that are useful across the entire image (due to the fact that image statistics are stationary). The pooling layers are responsible for reducing the sensitivity of the output to slight input-shift and distortions and increasing the reception field for later layers. Since 2012, one of the most notable results in deep learning is the use of CNNs to obtain a remarkable improvement in the ImageNet classification challenge [21,22]. Based on this technological breakthrough in image classification, various improvements have been proposed for the network architectures in vision models [23,24,25]. Although ANNs have been remarkably successful in many applications, including object detection [26,27], image segmentation [28,29,30], and action recognition [31,32], they are still limited in the way they deal with temporal information.

## 4. SNN Models

**Figure 2:**
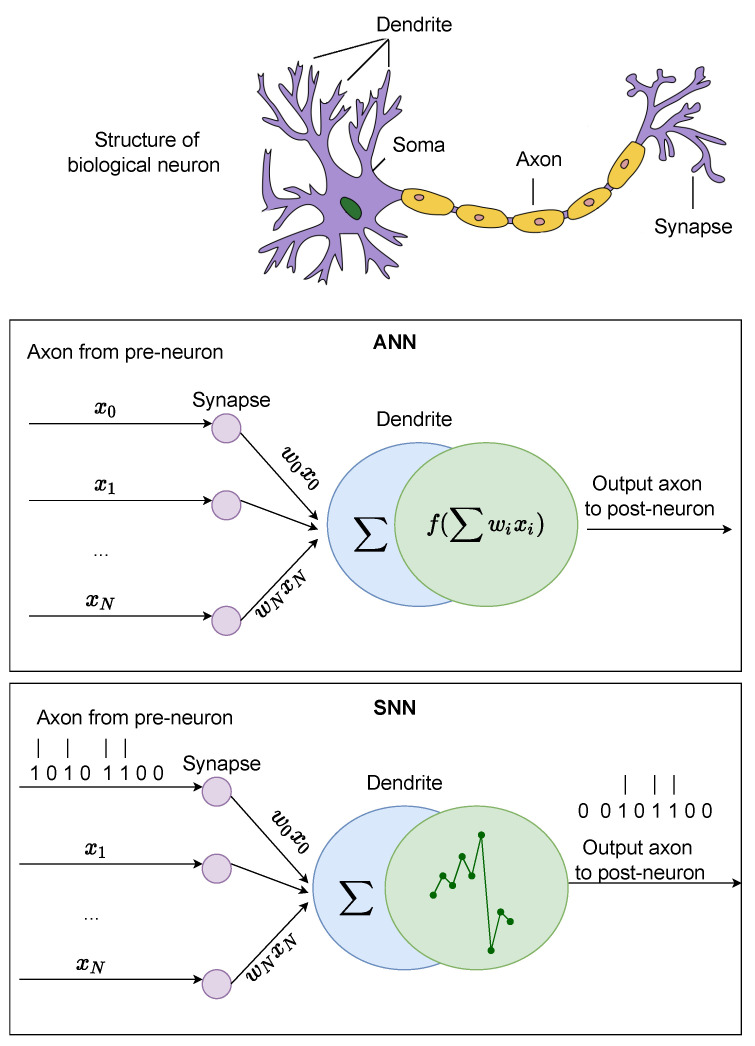
A comparison between the biological neuron, artificial neuron, and spiking neuron.

The ability to simultaneously record the activity of multiple cells has led to the idea that the time difference between spikes in different neurons and the spike timing itself can have functional significance. Since the firing rate model cannot handle the problem of this perspective, a model describing the timing of spikes and the variation of the sub-threshold membrane potential has been investigated. A model that handles the generation of such spikes is distinguished from the firing rate model and called the spiking model. Such neuron models are generally expressed in the form of ordinary differential equations. Figure 2 depicts the differences between the biological neuron, artificial neuron, and spiking neuron.

### 4.1. Spiking Neuron Models

**Figure 3:**
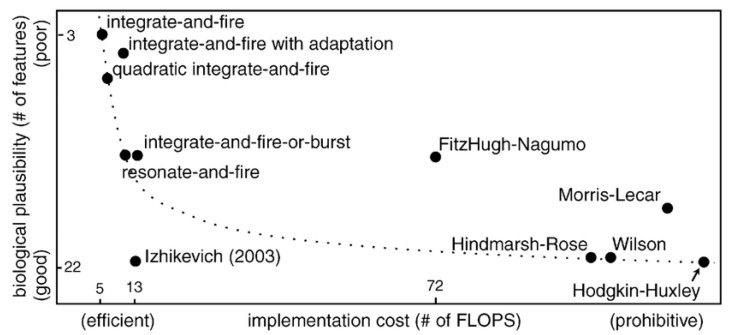
A comparison of spiking neuron models in terms of implementation cost and biological plausibility (adopted from [33]).

A variety of spiking neuron models have been proposed, and they display trade-offs between biological accuracy and computational feasibility (Figure 3). Choosing an appropriate model depends on the user requirements. Spike-based neuron models are reviewed regarding the computational efficiency and biological plausibility in [33]. In this section, several models of spiking neurons are presented.

#### 4.1.1. Hodgkin–Huxley (HH) Model

Hodgkin and Huxley conducted the experiment on the giant axon of a squid and concluded that two types of ion channels, K${}^{+}$ channel and Na${}^{+}$ channel, are involved in the generation of the action potential [34]. This model can be expressed by adding two terms that take care of the behavior of those two ion channels to Equation (9). Although the change in permeability of the ion channel is actually due to the structural change of the protein, it can be described phenomenologically by the analogy of opening and closing the gates.

(5) $$C_{m}\frac{dv_{m}\left(t\right)}{dt}=I_{ion}\left(t\right)+I_{syn}\left(t\right)$$

(6) $$\begin{array}{cc} I_{ion}\left(t\right) & =G_{K}n^{4}\left(v_{m}-E_{K}\right)+G_{Na}m^{3}h\left(v_{m}-E_{Na}\right)+G_{L}\left(v_{m}-E_{L}\right) \end{array}$$

where $C_{m}$ is membrane capacitance (pF), $v_{m}$ is the membrane potential (mV), $I_{syn}$ is synaptic input current (pA), $G_{K}$ represents conductance of K${}^{+}$ ion, $E_{K}$ represents reversal potential of K${}^{+}$ ion, $G_{Na}$ represents conductance of Na${}^{+}$ ion, $E_{Na}$ represents reversal potential of Na${}^{+}$ ion, $G_{L}$ represents leak conductance, and $E_{L}$ represents leak reversal potential, which is now thought to be a Cl${}^{-}$ ion’s reversal potential. *n*, *m*, and *h* are dimensionless quantities between zero and one that are associated with potassium channel activation, sodium channel activation, and sodium channel inactivation, respectively.

The three gates, *n*, *m*, and *h*, are described by the following differential equation, where *g* represents the gating variables *n*, *m*, and *h*, and the transition rate (where $\alpha_{g}\left(v\right)$ is the transition rate from non-permissive to permissive states, whereas $\beta_{g}\left(v\right)$ is the transition rate from permissive to non-permissive states) for each gate $\alpha_{g}\left(v\right)$ and $\beta_{g}\left(v\right)$ are defined in Equation (8) (in neural simulation software packages, the rate constants in Hodgkin–Huxley models are often parameterized using a generic functional form [35]: $\frac{A+Bv_{m}}{C+H\exp(\frac{v_{m}+D}{F})}$).

(7) $$\frac{dg}{dt}=\alpha_{g}\left(v_{m}\right)\left(1-g\right)-\beta_{g}\left(v_{m}\right)g$$

(8) $$\begin{array}{cc} \alpha_{m}\left(v_{m}\right) & =\frac{0.1(25-v_{m})}{\exp(\left(25-v_{m}\right)/10)-1}\\ \beta_{m}\left(v_{m}\right) & =4\exp\left(-v_{m}/18\right)\\ \alpha_{h}\left(v_{m}\right) & =0.07\exp\left(-v_{m}/20\right)\\ \beta_{h}\left(v_{m}\right) & =\frac{1}{\exp(\left(30-v_{m}\right)/10)+1}\\ \alpha_{n}\left(v_{m}\right) & =\frac{0.01(10-v_{m})}{\exp(\left(10-v_{m}\right)/10)-1}\\ \beta_{n}\left(v_{m}\right) & =0.125\exp\left(-v_{m}/80\right) \end{array}$$

By solving these equations, the Hodgkin–Huxley model can simulate the membrane potential behavior during spike generation without introducing spike generation procedures presented in the LIF model (Equation (10)). Although the Hodgkin–Huxley model is biologically accurate (the model is limited in the way that it only describes the channels and flow of ions in the neuron when generating spikes; several drawbacks have been pointed out [36,37]), it demands large computational resources and is infeasible in large-scale simulations.

#### 4.1.2. Leaky Integrate and Fire (LIF) Model

The model in which the input current is integrated over time until the membrane potential reaches a threshold without taking into account the biological ion channel behavior is called the integrate-and-fire (IF) model. The leaky integrate-and-fire (LIF) model reflects the diffusion of ions that occurs through the membrane when some equilibrium is not reached in the cell by introducing a “leak” term to the IF model. Because of its simplicity and low computational cost, the LIF model and its variants are one of the widely used instances of the spiking neuron model. The model dynamics are represented by the following equation:

(9) $$\begin{array}{c} C_{m}\frac{dv_{m}}{dt}=-G_{L}\left(v_{m}-E_{L}\right)+I_{syn}\left(t\right)\\ \mathrm{if}\;\;v_{m}\geq v_{\theta },\;\;v_{m}\leftarrow v_{peak}\;\mathrm{then}\;v_{m}\leftarrow v_{reset} \end{array}$$

where $v_{\theta }$ is the threshold voltage, $v_{peak}$ is the action potential, and $v_{reset}$ is the resetting membrane potential. When the voltage reaches the threshold $v_{\theta }$, usually one is used for simplicity, the neuron fires the spike, and then the voltage is reset to zero for a refractory period $\tau_{ref}$ that limits the firing frequency of a neuron.

When the synaptic input current is constant ($I_{syn}\left(t\right)=I$) and $v_{reset}=0$, we can solve for the membrane potential as follows:

(10) $$v_{m}\left(t\right)=R_{m}I\left(1-\exp\left(-\frac{t}{\tau_{m}}\right)\right)$$

where $R_{m}$ is the membrane resistance (MΩ), $\tau_{m}=R_{m}C_{m}$ is the membrane time constant. Since the neuron fires the spike when the membrane potential reaches the threshold, the first spike time $t^{\left(1\right)}$ can be found by setting $v_{m}\left(t\right)=v_{\theta }$:

(11) $$t^{\left(1\right)}=\tau_{m}\ln\frac{R_{m}I}{R_{m}I-v_{\theta }}$$

Therefore, steady-state firing rate can be found as:

(12) $$f={\left(\tau_{ref}+\tau_{m}\ln\frac{R_{m}I}{R_{m}I-v_{\theta }}\right)}^{-1}$$

Theoretically, it is possible to train a deep neural network using Equation (12) as the static non-linearity and make a reasonable approximation of the network in spiking neurons [38]. Intuitively, especially when $\tau_{ref}=0,\tau_{m}=1,R_{m}=1,$ and $v_{\theta }=1$, the firing rate of the neuron corresponding to the input current behaves similar to the ReLU activation function in ANNs. This feature is often utilized for ANN-to-SNN conversion.

#### 4.1.3. Izhikevich Model

Izhikevich proposed a model that combines the biological plausibility of the HH model’s dynamics and the computational efficiency of the LIF neurons [39]. The model is represented by the two-dimensional (2D) system of ordinary differential equations, and the Izhikevich model [40] can be expressed in the following form:

(13) $$C_{m}\frac{dv_{m}}{dt}=k\left(v_{m}-E_{L}\right)\left(v_{m}-v_{t}\right)-u+I_{syn}\left(t\right)$$

(14) $$\frac{du(t)}{dt}=a\left(b\left(v_{m}-E_{m}\right)-u\right)$$

with the auxiliary after-spike resetting

(15) $$\mathrm{if}\;v_{m}\geq v_{\theta }\;\mathrm{then}\;\left\{\begin{array}{c} v_{m}\leftarrow c\\ u\leftarrow u+d \end{array}\right.$$

where *u* represents the activation of K${}^{+}$ ionic currents and inactivation of Na${}^{+}$ ionic currents (pA), and $v_{t}$ is the instantaneous threshold potential (mV).

The Izhikevich model can exhibit the firing patterns of all known types of cortical neurons with the choice of parameters based on [40].

#### 4.1.4. Adaptive Exponential Integrate-and-Fire (AdEx) Model

The aforementioned Izhikevich neuron can be considered to be an adaptive quadratic integrate-and-fire model, whereas the adaptive exponential integrate-and-fire (AdEx) model [41] has an exponential voltage dependence, coupled with a slow variable, which models threshold adaptation as follows:

(16) $$\begin{array}{cc} C_{m}\frac{dv_{m}}{dt} & =-G_{L}\left(v_{m}-E_{L}\right)\\ & +G_{L}\Delta_{T}\exp\frac{v_{m}-v_{T}}{\Delta_{T}}-w+I_{syn} \end{array}$$

(17) $$\tau_{w}\frac{dw}{dt}=a\left(v_{m}-E_{L}\right)-w$$

with reset conditions

(18) $$\mathrm{if}\;v_{m}\geq v_{\theta }\;\mathrm{then}\;\left\{\begin{array}{c} v_{m}\leftarrow v_{reset}\\ w\leftarrow w+b \end{array}\right.$$

where *w* is the slow variable taking into account adaptation, $V_{T}$ is the rheobase current, $\Delta_{T}$ models the sharpness of the Na${}^{+}$ channels’ activation function.

The LIF model can be obtained from the AdEx model by taking the limit $\Delta_{T}\to 0$ and removing the adaptation current *w*. The AdEx model shares the ability to reproduce firing patterns at a low computational cost such as the Izhikevich neuron.

### 4.2. Synaptic Models

A synaptic interaction can be modeled as a process of binding a neurotransmitter to a closed receptor, which consequently opens it, and unbinding the transmitter from the receptor closing it. These can be modeled as a rate of ion channel opening or a variation of the conductance, as in the Hodgkin–Huxley model. Synaptic kinetics is defined by the number of neurotransmitters released from the pre-synaptic cell, the number of neurotransmitters bonded to the post-synaptic cell, or the opening rate of the ion channel of the post-synaptic cell. The following models are used to model the post-synaptic current (PSC) as well as the post-synaptic potential (PSP).

#### 4.2.1. Single Exponential Model

Assuming the binding of neurotransmitters is instantaneous, the behavior of PSC can be modeled as an exponential decay with a time constant. This can be modeled as:

(19) $$\begin{array}{c} f\left(t\right)=\exp\left(\frac{-(t-t_{k})}{\tau_{d}}\right)\\ s_{syn}\left(t\right)=\sum_{t_{k}<t}f\left(t-t_{k}\right) \end{array}$$

where $s_{syn}$ is synaptic kinetics, $t_{k}$ is the *k*th spike occurrence timing, and $\tau_{d}$ is synaptic decay time constant.

The previous equation can be expressed in a differential equation form:

(20) $$\frac{ds_{syn}}{dt}=-\frac{s_{syn}}{\tau_{d}}+\frac{1}{\tau_{d}}\sum_{t_{k}<t}\delta \left(t-t_{k}\right)$$

where δ(·) is the Dirac delta function that represents the occurrence of a spike.

#### 4.2.2. Double Exponential Model

While ignoring the physiological process, the double exponential model reproduces the behavior of the post-synaptic current (PSC) well, considering not only decay but the rise of the PSC.

(21) $$\begin{array}{c} f\left(t\right)=A\left(\exp\left(-\frac{t}{\tau_{d}}\right)-\exp\left(-\frac{t}{\tau_{r}}\right)\right)\\ A=\frac{\tau_{d}}{\tau_{d}-\tau_{r}}{\left(\frac{\tau_{r}}{\tau_{d}}\right)}^{\frac{\tau_{r}}{\tau_{r}-\tau_{d}}} \end{array}$$

where *A* is the normalizing constant and $\tau_{r}$ is the synaptic rising time constant.

The double exponential model can be expressed in a form of differential equations as follows:

(22) $$\begin{array}{cc} \; & \frac{ds_{syn}}{dt}=-\frac{s_{syn}}{\tau_{d}}+h\\ \; & \frac{dh}{dt}=-\frac{h}{\tau_{r}}+\frac{1}{\tau_{r}\tau_{d}}\sum_{t_{k}<t}\delta \left(t-t_{k}\right) \end{array}$$

where *h* is the helping variable. When $\tau =\tau_{d}=\tau_{r}$, Equation (21) is called the alpha function With these synaptic models, the input current to the cell $I_{syn}$ can be expressed as follows if we consider $s_{syn}$ as the pre-synaptic kinetics:

(23) $$I_{syn}\left(t\right)=Ws_{syn}\left(t\right)$$

where $s_{syn}\in R^{N_{pre}}$, $I_{syn}\in R^{N_{post}}$ is synaptic input of post-synaptic neurons, and $W\in R^{N_{post}\times N_{pre}}$ is the weight matrix that represents the synaptic strength between the pre- and post-synaptic neurons.

## 5. SNN Learning Mechanisms

Learning in neural networks involves the modification of the connectivity of neurons. Unlike the ANNs, which can be successfully trained by stochastic gradient descent and backpropagation, SNNs still do not have solid training methods. The native training methods of SNNs can be classified into: supervised learning with gradient descent and spike backpropagation, unsupervised learning with local learning rule at the synapse (e.g., spike-time-dependent plasticity), and reinforcement learning with reward/error signal using reward modulated plasticity. Synaptic plasticity is the biological process by which specific patterns of synaptic activity result in changes in synaptic strength. Synaptic plasticity was first proposed as a mechanism for learning and memory on the basis of theoretical analysis by [42]. Although the local learning rule at the synapse is said to be biologically more plausible, the learning performance is usually lower than that of supervised learning. An alternative approach to indirectly train the SNNs is the conversion of ANNs to SNNs [43]. Among those methods, state-of-the-art results are mostly obtained from the model conversion from ANNs.

### 5.1. Spike-Based Backpropagation

Similar to the backpropagation algorithm for ANNs, SpikeProp [44] is designed to determine a set of the desired firing timings of all output neurons at the post-synaptic neurons for a given set of the input pattern. Event-based methods, including SpikeProp, have the derivative term defined only around the firing time, whereas [45,46,47] ignore the temporal effect of the spike signal. Reference [48] proposed an improved method of SpikeProp called SuperSpike that utilizes the derivative of the membrane potential instead of the spike, which allows training a model with an absence of spike occurrence. SuperSpike uses the van Rossum distance [49] between the output and desired spike trains as the loss function, while SpikeProp uses the sum-squared error. The following shows the loss function for the network in time interval t∈[0,T].

(24) $$L=\frac{1}{2}\int_{0}^{T}{\left(\alpha \times \left(s\left(t\right)-\hat{s}\left(t\right)\right)\right)}^{2}dt$$

where α is a normalized smooth temporal convolution kernel, s is the output spike train, and $\hat{s}$ is a target spike train. Here, spike train is represented as $s\left(t\right)=\sum_{t_{k}<t}\delta \left(t-t_{k}\right)$.

When calculating the derivative of Equation (24) with respect to the synaptic weights, the problematic term $\frac{\partial s}{\partial w}$ that contains the Dirac delta function appears. In order to avoid this term, the spike train is approximated with a continuous auxiliary function of the membrane potential of the LIF model.

(25) $$\frac{\partial s}{\partial w}\approx \frac{\partial \sigma (v_{m})}{\partial w}=\sigma^{\prime }\left(v_{m}\right)\frac{\partial v_{m}}{\partial w}$$

where σ(x)=x/(1+|x|) represents a fast sigmoid. Here we further approximate $\frac{\partial v_{m}}{\partial w}\approx \epsilon \times s$ with a normalized smooth temporal convolution kernel ϵ.

(26) $$\frac{\partial L}{\partial w}=\int_{0}^{T}\alpha \times \left(s-\hat{s}\right)\;\alpha \times \left(\sigma^{\prime }\left(v_{m}\right)\left(\epsilon \times s^{pre}\right)\right)dt$$

where the $\alpha \times (s-\hat{s})$ is an error signal and $\alpha \times (\sigma^{\prime }\left(v_{m}\right)\left(\epsilon \times s^{pre}\right))$ is a synaptic eligibility trace.

SLAYER [50] distributes the credit of error back in time in order to solve the drawback of event-based methods. SLAYER assumes a stochastic spiking neuron approximation for the IF model with a refractory response and can simultaneously learn both synaptic weights and axonal delays.

(27) $$\frac{\partial L}{\partial w}=\int_{0}^{T}\rho \left(t\right)\left(\alpha ⊙e\right)\left(\alpha \times s^{pre}\right)dt$$

where ρ(t) is the probability density function that could be formulated with the spike escape rate function [51], ⊙ represents the element-wise correlation in time, and *e* is the backpropagation estimate of error.

### 5.2. Spike-Time-Dependent Plasticity (STDP)

Spike-time-dependent plasticity (STDP) is an unsupervised Hebbian learning mechanism, which adjusts synaptic weight based on the temporal order of the pre- and post-synaptic spikes [52,53]. When the pre-synaptic spike arrives before a post-synaptic spike, the synaptic weight is increased, which is known as long-term potential (LTP). If the arrival timing of the synaptic spike is reversed, the synaptic weight is decreased, which is known as long-term depression (LTD).

(28) $$\Delta w=\left\{\begin{array}{cc} A_{+}\exp\left(\frac{t_{pre}-t_{post}}{\tau_{+}}\right) & \mathrm{if}\;t_{pre}\leq t_{post}\\ -A_{-}\exp\left(-\frac{t_{pre}-t_{post}}{\tau_{-}}\right) & \mathrm{if}\;t_{pre}>t_{post} \end{array}\right.$$

Equation (28) suggests that the synaptic strength can be increased or decreased infinitely, which is biologically unrealistic and makes learning unstable. Biological neurons have a capacity to regulate their own excitability relative to network activity by decreasing the strength of each synapse so that the relative synaptic weighting of each synapse is preserved [54]. This phenomenon is called homeostatic scaling and can be implemented by making $A_{\pm }$ weight dependent. With the following exponential rule, the magnitude of the weight modification is regularized according to the current synaptic weight.

(29) $$\left\{\begin{array}{c} A_{+}\left(w\right)=\eta_{+}\exp\left(w_{init}-w\right)\\ A_{-}\left(w\right)=\eta_{-}\exp\left(w-w_{init}\right) \end{array}\right.$$

Here, $\eta_{\pm }$ are learning rates that take small positive values, and $w_{init}$ refers to the initial weight of the synapse (sometimes this term is dropped).

**Figure 4:**
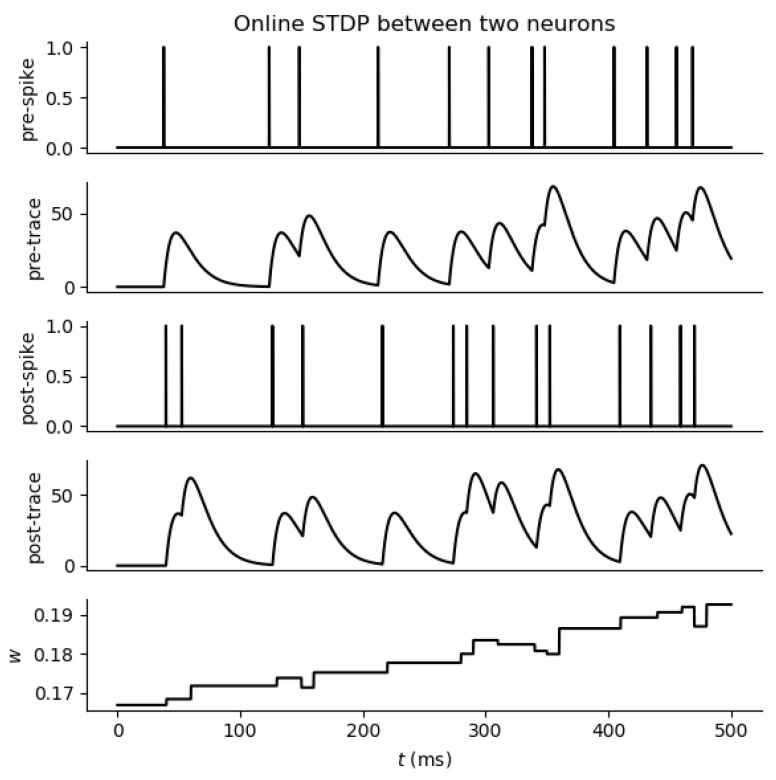
Weight change in two neurons based on STDP learning rule.

In terms of biology as well as the implementation, it is infeasible to remember all the times of spike occurrence, as seen in Equation (28). This is where the spike trace *x* is introduced:

(30) $$\frac{dw}{dt}=A_{+}x_{pre}\cdot \delta_{post}-A_{-}x_{post}\cdot \delta_{pre}$$

(31) $$\begin{array}{c} \frac{dx_{pre}}{dt}=-\frac{x_{pre}\left(t\right)}{\tau_{+}}+\delta \left(t\right)\\ \frac{dx_{post}}{dt}=-\frac{x_{post}\left(t\right)}{\tau_{-}}+\delta \left(t\right) \end{array}$$

where $\tau_{+}$ and $\tau_{-}$ are the time constants. Figure 4 shows the response of a spike trace and corresponding weight modifications based on STDP. The spike trace $x_{pre}$ can be interpreted as an opening rate of N-methyl-D-aspartate (NMDA) receptor and $x_{post}$ as $Ca^{2+}$ influx through voltage-gated $Ca^{2+}$ channels activated by a backpropagating action potential (bAP). This multiplicative STDP implementation that is inherently stable by combining the weight-dependent exponential rule with spike trace information is often referred to as stable STDP (S-STDP) [55].

In the following subsections, we will review various STDP variants.

#### 5.2.1. Anti-Hebbian STDP (aSTDP)

Although STDP-like synaptic weight modifications have been found in various neuronal systems, all the systems do not follow the STDP rule. Synapses between parallel fibers and Purkinje-cells in the cerebellum-like structure, for example, follow an anti-Hebbian temporal order [56]. The anti-Hebbian STDP (aSTDP) shows the opposite dependence on the relative timing of pre-synaptic input and the post-synaptic spike compared to STDP. With aSTDP, pre-synaptic activity occurring before post-synaptic activity leads to depression, and vice versa. The aSTDP rule is given:

(32) $$\Delta w=\left\{\begin{array}{cc} A_{+}\exp\left(-\frac{t_{pre}-t_{post}}{\tau_{+}}\right) & \mathrm{if}\;t_{pre}>t_{post}\\ -A_{-}\exp\left(\frac{t_{pre}-t_{post}}{\tau_{-}}\right) & \mathrm{if}\;t_{pre}\leq t_{post} \end{array}\right.$$

Compared to the standard STDP, the directions of the greater than/less than signs is opposite, and the magnitude of the learning rate could be different from that for STDP.

#### 5.2.2. Mirrored STDP (mSTDP)

Mirrored STDP was introduced as an effort to implement autoencoders in a biologically realistic fashion [57]. mSTDP combines STDP and aSTDP for feedforward and feedback connections of a two-layer autoencoder such that feedforward and feedback learning is symmetric. This learning rule accounts for high LTP correlation with no causality. However, the biological plausibility is limited because it neglects the causality underlined by Hebb [42].

#### 5.2.3. Probabilistic STDP

A probabilistic variant of simplified STDP [58] that adjusts the synaptic weight for LTP according to an exponential function of the current weight magnitude was introduced by [59]. Probabilistic STDP shows the robustness in performance regardless of a complexity in the spiking neuron model, i.e., non-leaky IF neurons and Izhikevich-like neurons.

(33) $$\Delta w=\left\{\begin{array}{cc} \eta_{+}\exp\left(-w\right) & \mathrm{if}\;t_{pre}\leq t_{post}\\ -\eta_{-} & \mathrm{if}\;t_{pre}>t_{post} \end{array}\right.$$

#### 5.2.4. Reward Modulated STDP (R-STDP)

While STDP operates based upon the correlation between the spike timings of the pre- and post-synaptic neurons, a reward signal is introduced to modulate STDP in order to implement a reinforcement learning mechanism. If the reward is positive, the corresponding synapse is potentiated; otherwise, the corresponding synapse is depressed. According to [60], dopaminergic neurons are characterized as having two different firing patterns. In the absence of any stimulus, they exhibit a slow (1–8 Hz) firing rate, known as background firing. When stimulated, the dopaminergic neurons exhibit burst firing. Burst firing is where neurons fire in very rapid bursts, followed by a period of inactivity. The modulation is conducted by introducing an eligibility trace *z* for pre- and post-synaptic spike occurrence as follows:

(34) $$\frac{dw}{dt}=\eta r\left(t\right)z_{i,j}\left(t\right)$$

(35) $$\frac{dz_{i,j}}{dt}=-\frac{z_{i,j}\left(t\right)}{\tau }+STDP\left(t\right)$$

where r(t) is the reward given at time *t*, *z* is the eligibility trace.

### 5.3. Prescribed Error Sensitivity (PES)

Prescribed error sensitivity (PES) is a supervised learning rule suited for online learning for adaptive control that learns a function by minimizing an external error signal frequently used with the neural engineering framework (NEF) [61]. This rule has been used for many works, including a biologically detailed neural model of hierarchical reinforcement learning [62] and adoptive control of quadcopter flight [63]. The weight update for this rule is defined as follows:

(36) Δw=ηe(t)a

where e(t) is an error signal at time *t*, and *a* is the rate activity of each neuron.

### 5.4. Intrinsic Plasticity

The intensity of an average synaptic input in the brain may change dramatically. Neurons maintain responsiveness to both small and large synaptic inputs by regulating intrinsic excitability to promote stable firing. This way, neuronal activity can keep from falling silent or saturating when the average synaptic input falls extremely low or rises significantly high. Intrinsic plasticity (IP) regulates the firing rate of a neuron within an appropriate range [64,65]. The firing rate entropy can be influenced by the neuron’s intrinsic properties. By changing these intrinsic properties, the neuron can achieve the optimal firing rate distribution.

(37) $$\phi =\left\{\begin{array}{cc} -\eta \exp(\frac{\tau_{min}-\Delta t_{ISI}}{\tau_{min}}) & \;\mathrm{if}\;\Delta t_{ISI}<T_{min}\\ \eta \exp(\frac{\Delta t_{ISI}-\tau_{max}}{\tau_{max}}) & \;\mathrm{if}\;\Delta t_{ISI}>T_{max}\\ 0 & \;\mathrm{otherwise} \end{array}\right.$$

(38) $$b\leftarrow b+b_{max}\cdot \phi$$

where η is a learning rate, $T_{min}$ and $T_{max}$ are thresholds that determine the desired range of inter-spike interval (ISI) represented as $\Delta t_{ISI}$.

During the training, the most recent ISI is examined and neuronal excitability is adjusted. When ISI is larger than the threshold $T_{max}$, the neuronal excitability is strengthened to make the neuron more sensitive to input stimuli, and if ISI is less than the threshold $T_{min}$, the neuronal excitability is weakened to make the neuron less sensitive to input stimuli.

### 5.5. ANN-to-SNN Conversion

Most ANN-to-SNN conversion methods have focused on converting ReLU to IF neurons. Reference [43] proposed an ANN-to-SNN conversion method that neglects bias and max-pooling. In subsequent work, reference [66] proposed data-based normalization to improve the performance in deep SNNs. Reference [67] presented conversion methods of batch normalization and spike max-pooling. Reference [68] expanded conversion methods to VGG and residual architectures. One core hypothesis of several ANN-to-SNN conversion designs is that the heavy computational cost of existing ANNs results from the continual transmission of real-valued activities between connected nodes in the network, as well as the subsequent matrix multiplication or convolution [69]. As a result, implementing ANN-to-SNN conversion may enable the same information transmission and function but decrease the costs of signal transmission and computation. Binary-valued spikes both reduce the number of bits per transmission by turning real-valued signals into binary ones, and they make signals sparse in time by not transmitting information for each connection every timestep. These ANN-to-SNN conversion methods are based on the idea of importing pre-trained parameters (e.g., weights and biases) from an ANN to an SNN. ANN-to-SNN conversion methods have achieved comparable results in deep SNNs to those of original ANNs (e.g., VGG and ResNet) and can be considered as a solution to the energy-efficiency problem of ANNs in the deployment time.

## 6. Spike Encoding

Since SNNs utilize the spike and spike sequences to convey the information, encoding real data into spikes is a substantial step in creating SNNs. Although the way information is encoded into spikes in biology is one of the biggest unresolved challenges in neuroscience. Two main encoding schemes, *rate encoding* and *temporal (pulse) encoding*, can be found in many kinds of literature. In addition, it is noteworthy that some sensors, such as Dynamic Vision Sensor (DVS), can produce raw spike sequences.

### 6.1. Rate Encoding

The rate encoding scheme is based on the average number of spikes over time; information is encoded with a number of spikes generated over a time window. Depending on the different averaging schemes, there are several ways to define the firing rate, such as an average over time or an average over several repetitions.

(39) $$n=\int_{0}^{T}dt\;\delta \left(t-t_{k}\right)$$

where *T* is the time window, and $t_{k}$ is the time of spike occurrence. Then, the firing rate *r* can be expressed as:

(40) $$r=\frac{n}{T}$$

This firing rate can be used as an input for rate-base neuron models, i.e., ANNs, where the activation function represents the frequency–current (FI) curve.

The firing rate can also be utilized to model the discrete spikes with the point process. In the Poisson process, which is one of the point processes, the probability of the random variable N(t) being equal to *n*, i.e., when the probability of a point occurring follows a Poisson distribution with intensity λ, the probability of a spike occurring *n* times by time *t*, is given by: $P\left\{N\left(t\right)=n\right\}=\frac{{\left(\lambda t\right)}^{n}}{n!}e^{-\lambda t}$. Then, the single spike occurrence during a short time step Δt is:

(41) $$P\left\{N\left(\Delta t\right)=1\right\}=\lambda \Delta t\left(e^{-\lambda \Delta t}\right)≃\lambda \Delta t+o\left(\Delta t\right)$$

where the $e^{-\lambda \Delta t}$ term is approximated with the McLaughlin expansion.

When encoding an image into spike sequence, we can assume each pixel value corresponds to the firing rate *r*, and following Equation (41), spike occurrence for each time step *t* can be obtained as:

(42) $$s=\left\{\begin{array}{cc} 1 & \mathrm{if}\;\xi ∼U(0,1)<r\Delta t\\ 0 & \mathrm{otherwise} \end{array}\right.$$

### 6.2. Temporal Encoding

The temporal encoding scheme is based on the exact timing of spikes, where the more salient information is encoded as earlier spike times. Compared to the rate encoding scheme, temporal encoding produces much sparser spikes since the spike-timing rather than the spike-frequency represents information. Although the temporal code allows representing the features of the input with small groups of neurons, it contains a vulnerability to input noise or temporal jitter.

When encoding an image, each individual pixel value ranging from 0 to 255 can be simply used to produce the spike time that is proportional to the brightness of the pixel. For instance, a pixel with normalized brightness of 0.1 corresponds to a spike time at t=0.1. In a grayscale image, white pixels (brightness equals 1 or 255) do not cause spikes, as it can be considered that they do not carry any information.

## 7. SNNs in Computer Vision

SNNs have been a driving factor in the development of many modern computer vision and other signal processing techniques. The application of SNNs is gradually being considered in computer vision where data consisting of temporal information is handled or where the saving of computational resources is aimed. The former case often involves the use of an event camera or LiDAR sensor whose data has importance in the temporal dimension. The latter case often focuses on the conversion of ANNs into SNNs so that deep neural network models can embrace the energy-efficient operations of neuromorphic hardware.

Although some studies have shown SNNs can be used for image classification on large datasets such as ImageNet [38,67,68], most applications of SNNs are still limited to less complex datasets such as MNIST [70], N-MNIST [71], and N-Caltech101 [71]. One of the primary reasons for the limited application scope is the complex dynamics and non-differentiable operations of spiking neurons. Recently, some remarkable studies have applied SNNs for object detection tasks [72,73,74], showing comparative results with DNNs while requiring much less energy for the computations. Following the successes of the ANNs to SNNs conversion methods on image classification and object detection tasks, reference [75] leveraged SiamFC [76] and introduced SiamSNN, a spike-based Siamese network for object tracking. Recently, UNet-based SNN in [69] leveraged the Nengo framework to translate a simplified U-Net into a spiking network to deploy on the Intel Loihi neuromorphic chip. The UNet-based SNN model is implemented with two frameworks: TensorFlow and NengoDL [77]. Furthermore, a partitioning algorithm, which minimizes inter-chip communication resulting in a faster and more energy-efficient network, is proposed in [69] to deploy SNN on Loihi.

Unlike frame-based cameras, event-based cameras are often referred to as bio-inspired silicon retinas. However, event-based cameras require a high temporal resolution (in the order of microseconds) and a fraction of power consumption. The combination of spiking neural networks and event-based vision sensors holds the potential of highly efficient and high-bandwidth optical flow estimation [55]. Reference [78] proposed Spike-FlowNet, a deep hybrid neural network architecture integrating SNNs and ANNs for efficiently estimating optical flow from sparse event camera outputs without sacrificing performance.

**Figure 5:**
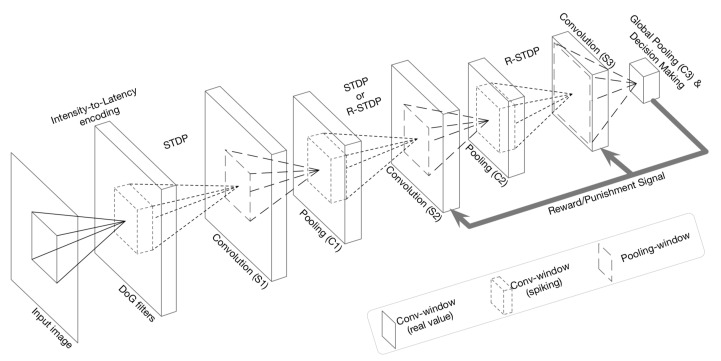
The overall structure of DCSNN for digit recognition. Courtesy of [79].

To illustrate how to implement an SNN framework for computer vision, we choose the task of image classification with the DCSNN network [79]. The overall structure of DCSNN for digit recognition is shown in Figure 5. The input image is convolved with six different Gaussian (DoG) filters at various scales with zero padding. Window sizes are set to 3 × 3, 7 × 7, and 13 × 13, where their standard deviations (σ1, σ2) are (3/9, 6/9), (7/9, 14/9), and (13/9, 26/9), respectively. Then, a spike is generated and propagated to the next layer by an intensity-to-latency encoding [80]. From the output of the DoG filters, all the values below 50 are ignored and the remaining values are descendingly sorted, denoting the order of spike propagation. Generated spikes are processed through three spiking convolution-then-pooling layers (S1–C1, S2–C2, and S3–C3). The convolutional layer (S-layer) contains several 2-dimensional grids of IF neurons, which constitute the feature maps. All S-layers are trainable, employing either STDP or R-STDP learning rules. The C-layer has the same number of feature maps as its previous S-layer, and there is a one-to-one association between maps of the two layers. There are two types of C-layers: spike-based and potential-based. The network makes its decision in C3, where neurons are pre-assigned to digits, and the decision is the digit assigned to the neuron with either the maximum internal potential or the earliest spike time. When the decision of the network has been made, it will be compared with the original label of the input image. By using the R-STDP rule for synaptic plasticity, a reward or punishment will be generated depending on if the decision and ground truth label match or mismatch [67].

Table 2 summarizes the use of SNNs in the field of computer vision.

## 8. SNNs in Robotic Control

Mobile robots with continuous high-dimensional observation and action spaces are increasingly being deployed to solve complex real-world tasks. Given their limited onboard energy resources, there is an unmet need to design energy-efficient solutions for the continuous control of these autonomous robots.

Biology shows that the event-based paradigm is applicable not just to perception and inference but also to control. Spiking neural networks have been utilized as a “brain” of robots that provides robotic perception and action to mimic the behaviors captured in nature. Most commonly, the utilization of SNNs in robotic applications involves hand-crafting and tuning for the task of interest. Many fields of robotics, e.g., locomotor systems, have been inspired by biological systems. Nowadays, several methods have been proposed to achieve locomotion in a variety of robots, which is known as a central pattern generator (CPG). CPG is a neural network in which interconnected excitatory and inhibitory neurons produce an oscillatory, rhythmic output without some rhythmic inputs. Most of the current research explores ANNs based on non-spiking neurons, but there is a growing body of research on SNNs. Reference [81] presented the first implementation of a real-time neuromorphic spiking CPG (sCPG) that runs on the SpiNNaker to command a hexapod robot to perform a walk, trot, or run motion. Reference [82] implemented sCPG with an AdEx neuron model that exhibits a tripod-like gait. Their model can manipulate the amplitude, frequency, and phase while the network is running, indicating that these characteristics can be updated in an online manner to further explore the role of sensory feedback in shaping locomotion.

In robotics, the lamprey has often been used as a model for understanding the role of CPG in locomotion. The lamprey swims by propagating a mechanical wave, transmitted along the body. Reference [83] proposed to implement a sCPG using an analog/digital VLSI device interfaced with an FPGA board, which can be directly interfaced to the actuators of a bio-mimetic robotic lamprey. Reference [84] used the sCPG model implemented in Nengo to produce the swimming gaits modulated by the high-level brainstem control of a simulated lamprey robot model in various scenarios. They showed that the robot can be controlled dynamically in direction and pace by modifying the input to the network.

Inspired by the success of SNNs on event-based cameras, reference [85] proposed, for the very first time, a fully embedded application of the Loihi neuromorphic chip prototype in a flying robot to bridge the gap between simulation and the real world. In this work, the SNN architecture is evolved in a highly randomized and abstracted vertical simulation and takes the ventral optic flow divergence as its input to determine the thrust setpoint to achieve a smooth landing. Focusing on proportional, integral, derivative (PID) controller in neuromorphic hardware, reference [86] improved the work in [87] and proposed an event-based PID controller to improve the PID controller on Loihi. In [86], they re-designed the integral path of the controller to cope with a limited resolution of value representation, which led to fast saturation of the I-path. Then, they simplified the network, removing the inner control loop and simplified the network, removing the inner control loop.

In addition to a pattern generator and motor control, navigation is an important task in robotics. With the requirement of energy efficiency in simultaneous localization and mapping (SLAM), which is crucial for mobile robots exploring unknown environments, SNN is an appropriate solution. Reference [88] proposed a biologically constrained SNN architecture to solve the unidimensional SLAM problem on Loihi. In [88], the robot’s heading is determined via spike-based recursive Bayesian inference of multisensory cues (i.e., visual and odometry information). Reference [89] demonstrated a model of rat hippocampus place, grid, and border cells implemented with the SpiNNaker. The implemented place cells were used to represent the location of landmarks for “home” and “cheese”, whereas the grid cells provide displacement information to the robot. They showed that the robot can detect these landmarks correctly. Reference [90] presented a brain-like navigation system with LIF neurons trained by STDP. In this work, reference [90] shows that SNN may robustly control an autonomous robot in mapping and exploring an unknown environment, while compensating for its own intrinsic hardware imperfections, such as partial or total loss of visual input. Reference [91] proposed a variant of deep deterministic policy gradient (DDPG), called spiking deep deterministic policy gradient (SDDPG), which consists of a spiking actor network and a deep critic network that were trained jointly using gradient descent for energy-efficient mapless navigation. This work explored an indirect SNN training approach based on the reward-modulated spike-timing-dependent plasticity (R-STDP) learning rule and supervised learning framework. The model was validated with Turtlebot2 platform and Intel’s Kapoho-Bay USB chipset. The authors claimed that the proposed method performed slightly better than the state-of-the-art thanks to the generalization introduced by the Poisson spike encoding of the state input.

**Figure 6:**
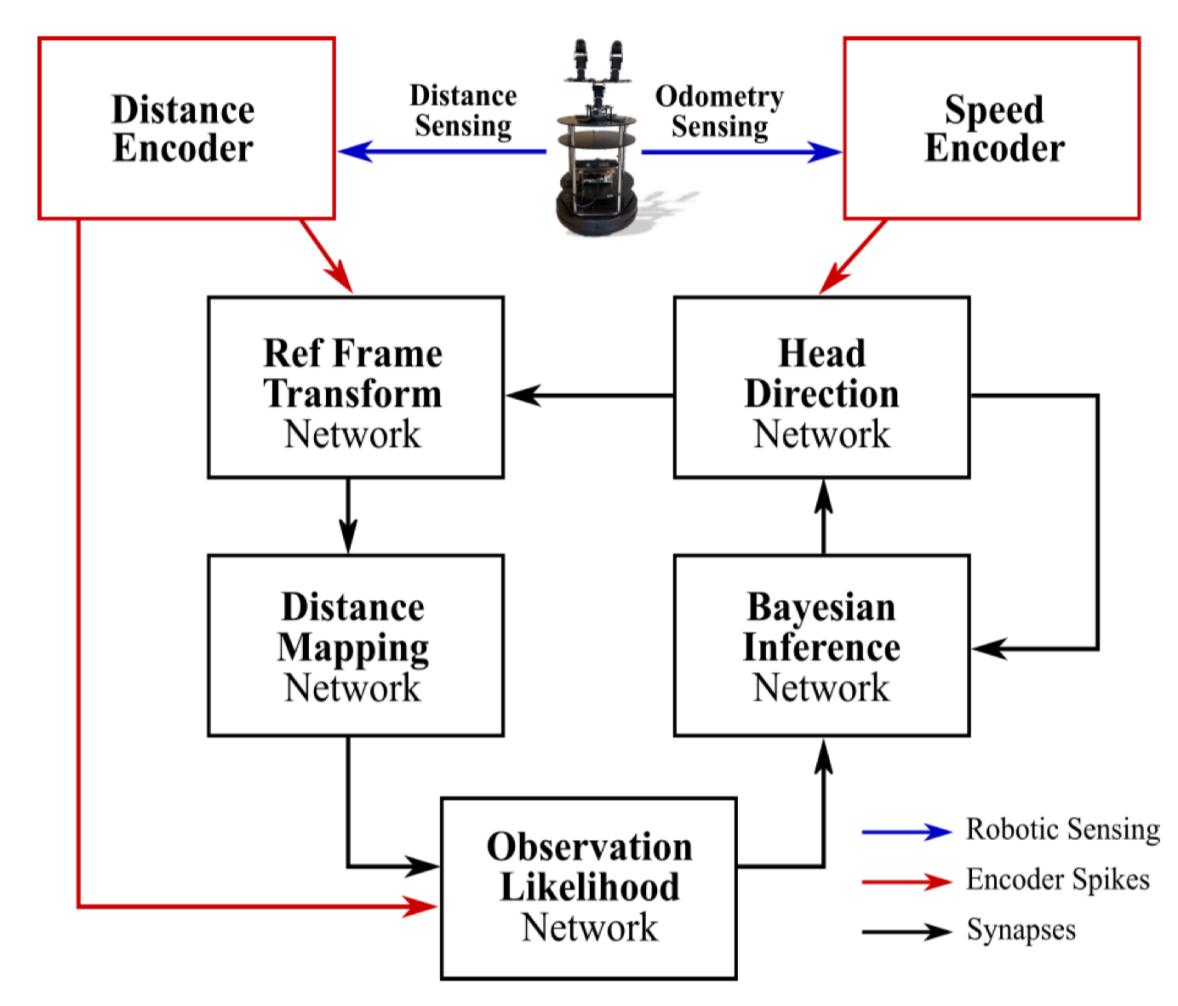
The overall structure of SNN architecture for SLAM. Courtesy of [88].

In this category, we will introduce [88] as an instance to show how SNNs are used in robotics. The model has two sensory spike rate-encoders and five sub-networks, as shown in Figure 6. The odometry sensor and the RGB-depth camera signals drive the neural activity of speed cells and sensory neurons encoding the angular speed and the distance to the nearest object, respectively. With five sub-networks, Head Direction (HD) receives the input from the speed cells and plays the role of the heading of the robot; Reference Frame Transformation (RFT) receives the egocentric input from sensory neurons and generates the allocentric distance representation in the world reference frame (defined by the HD network); Distance Mapping (DM) learns the allocentric observations from the RFT and forms a map of the robot’s surrounding environment; Observation Likelihood (OL) uses the map from the DM to compute the observation likelihood distribution of the robot’s heading based on the egocentric observation from sensory neurons; Bayesian Inference (BI) produces a near-optimal posterior of the robot’s heading and corrects the heading representation within the HD.

Table 3 summarizes the use of SNNs in the field of robotics.

## 9. Available Software Frameworks

The steadily increasing interest in SNN has led to many attempts to develop SNN libraries for Python. Unlike ANNs, the objectives in SNNs are time consumption and energy efficiency. To provide functional systems for researchers to execute applications that are designed with SNNs, several software frameworks have been proposed to provide SNN platforms. We provide a list of open-source software frameworks for the SNN simulation with some emphasis on the relation with deep learning frameworks in Table 4.

## 10. Conclusions and Future Perspectives

In this paper, we present a review of the fundamentals of spiking neural networks (SNNs) and provide a survey of the literature on the use of SNNs in computer vision and robotics applications, which demonstrates the great potential of SNNs in the research community. Over the past decade, SNNs have gained huge attention and shown they are promising in temporal information processing capability, low power consumption, and high biological plausibility. However, realizing the full potential of SNNs requires solving several challenges ahead of us.

- *Training of SNNs*: There are two main approaches to train SNNs: (i) training SNNs directly based on either supervised learning with gradient descent or unsupervised learning with STDP (ii) convert a pre-trained ANN to an SNN model. The first approach has the problem of gradient vanishing or explosion because of a non-differentiable spiking signal. Furthermore, an SNN trained by gradient descent is restricted to shallow architectures and produces low performance on large-scale datasets such as ImageNet. The second approach increases the computational complexity because of the large number of timesteps, even though these SNNs achieve comparable accuracy to ANNs, due to the similarity between SNNs and recurrent neural networks (RNNs), and results in backpropagation through time (BPTT). Recently, reference [92] showed that RTRL, an online algorithm to compute gradients in RNNs, can be combined with an LIP neuron to provide good performance with a low memory footprint.
- *SNNs Architecture*: While the majority of existing works on SNNs have focused on the image classification problem and utilize available ANN architectures such as VGG or Resnet, having an appropriate SNN architecture is critical. Recently, meta-learning such as neural architecture search (NAS) has been utilized to find the best SNN architecture [93].
- *SNNs Performance on Large-scale Data*: While SNNs have shown an impressive advantage with regard to energy efficiency, their accuracy performances are still low compared to ANNs on large-scale datasets such as ImageNet. Recently, references [94,95,96] utilized the huge success of ResNet in ANNs to train deep SNNs with residual learning on ImageNet.

**Table 2:** Summary of recent applications of SNNs in computer vision.

Method Year	Training Paradigm	Description	Performance
Image Classification			
DCSNN [79] (2018)	STDP and RSTDP	A convolutional SNN was trained by a combination of STDP for the lower layers and reward-modulated STDP for the higher ones, which allows training the entire network in a biologically plausible way in an end-to-end fashion.	They have achieved 97.2% accuracy on the MNIST dataset. https://github.com/miladmozafari/SpykeTorch (accessed on 5 September 2021)
LM-SNNs [97] 2020	STDP	Lattice map spiking neural network (LM-SNN) model with modified STDP learning rule and biological inspired decision-making mechanism was introduced. Learning algorithm in LM-SNN manifests an unsupervised learning scheme.	Dataset: MNIST handwritten digits and a collection of snapshots of Atari Breakout.
Zhou et al. [98] (2020)	STDP	First work to apply SNNs to a medical image classification task. Utilized an STDP-based convolutional SNN to distinguish melanoma from melanocytic nevi.	Dataset: ISIC 2018 [74] includes 1113 images of MEL and 6705 images of NV. Performance: Without feature selection: 83.8% With feature selection: 87.7%
Zhou et al. [99] (2020)	R-STDP	An imbalanced reward coefficient was introduced for the R-STDP learning rule to set the reward from the minority class to be higher than that of the majority class and to set the class-dependent rewards according to the data statistic of the training dataset.	Dataset: ISIC 2018. Performance: classification rate of the minority class from 0.774 to 0.966, and the classification rate of the majority class is also improved from 0.984 to 0.993.
Lou et al. [100] (2020)	STDP	Both temporal and spatial characteristics of SNN are employed for recognizing EEG signals and classifying emotion states. Both spatial and temporal neuroinformatic data to be encoded with synapse and neuron locations as well as timing of the spiking activities.	74%, 78%, 80% and 86.27% for the DEAP dataset, and the overall accuracy is 96.67% for the SEED dataset
Object Detection			
Spiking YOLO [73] (2019)	ANN-to-SNN Conversion	Spiking-YOLO was presented for the first kind to perform energy-efficient object detection. They proposed channel-wise normalization and signed neuron with imbalanced threshold to convert leaky-ReLU in a biologically plausible way.	They have achieved mAP% 51.83 on PASCAL VOC and mAP% 25.66 on MS COCO
Deep SCNN [74] (2020)	Backpropagation	SNN based on the Complex-YOLO was applied on 3D point-cloud data acquired from a LiDAR sensor by converting them into spike time data. The SNN model proposed in the article utilized the IF neuron in spike time form (such as the one presented in Equation (11)) trained with ordinary backpropagation.	They obtained comparative results on the KITTI dataset with bird’s-eye view compared to Complex-YOLO with fewer energy requirements. Mean sparsity of 56.24% and extremely low total energy consumption of 0.247 mJ only.
Image Classification			
FSHNN [101] (2021)	STDP & SGD	Monte Carlo dropout methodology is adopted to quantify uncertainty for FSHNN and used to discriminate true positives from false positives.	FSHNN provides better accuracy compared to ANN-based object detectors (on MSCOCO dataset) while being more energy-efficient. Features from both unsupervised STDP-based learning and supervised backpropagation-based learning are fused. It also outperforms ANN, when subjected to noisy input data and less labeled training data with a lower uncertainty error.
Object Tracking			
SiamSNN [75] (2020)	ANN-to-SNN Conversion	SiamSNN, the first SNN for object tracking that achieves short latency and low precision loss of the original SiamFC was introduced along with an optimized hybrid similarity estimation method for SNN.	OTB-2013, OTB-2015, and VOT2016. SiamSNN achieves 50 FPS and extremely low energy consumption on TrueNorth.
Two multi-layered SNNs [102] (2020)	R-STDP	This addressed the issue of SNN-based moving-target tracking on a wheel-less snake robot. A Dynamic Vision Sensor (DVS) is utilized to perceive the target and encode it as spikes that are fed into the SNN to drive the locomotion controller of the snake robot.	The simulation experiments conducted in the NRP. Compared to SNN, the relative direction of the target to the robot is with less fluctuation when using the multi-layered SNN.
Object Segmentation			
Unet-based SNN [69] (2021)	ANN-to-SNN Conversion	Instead of using a fixed firing rate target for all neurons on all examples, Unet-based SNN regularizes a rank-based statistic computed across a neuron’s firing rates on multiple examples to allow a range of firing rates. Unet-based SNN also proposes the percentile-based loss function to regularize the (almost) maximum firing rate of each neuron across all examples. During the forward pass, it uses a modification of the ReLU non-linearity	Even achieve lower accuracy performance (92.13%) compared to Unet baseline (94.98% on Tensorflow and 92.81% on NengoDL) on the ISBI 2D EM Segmentation dataset, Unet-based SNN runs on the Loihi neuromorphic hardware with greater energy efficiency.
SpikeMS [103] (2021)	Backpropagation	SpikeMS includes spike counts and classification labels to address the problem of motion segmentation using the event-based DVS camera as input	SpikeMS achieves performance comparable to an ANN method but with 50× less power consumption on EV-IMO, EED and MOD datasets.
Chen et al. [104] (2021)	ANN-to-SNN conversion	Temporal redundancy between adjacent frames is capitalized to propose an interval reset method where the network state is reset after a fixed number of frames.	It achieved a 35.7× increase in convergence speed with only 1.5% accuracy drop using an interval reset of 20 frame
Image Classification			
SpikeSEG [105] (2021)	STDP & backpropagation	This is a spiking fully convolutional neural network used for semantic event-based image segmentation through the use of active pixel saliency mapping. Both spike-based imaging and spike-based processing are utilized to deliver fast and accurate class segmented spiking images.	The SpikeSEG network performs well on the synthetic dataset with accuracy values of 97% and mIoU of 74%
Optical-Flow Estimation			
Hierarchical cuSNN [55] (2019)	Stable STDP	The selectivity of the local and global motion of the visual scene emerges through STDP from event-based stimuli. The input statistics of event-based sensors are handled by an adaptive spiking neuron model. The neuron is learnt by the proposed stable STDP. The neuron model and STDP rule are combined in a hierarchical SNN architecture to capture geometric features, identify the local motion of these features, and integrate this information into a global ego-motion estimate.	It is evaluated on synthetic and real event sequences with the Event Camera Dataset on DAVIS and SEES1 DVS sensors. Code available: https://github.com/tudelft/cuSNN (accessed on 4 September 2021)
Spike-FlowNet [78] (2020)	Backpropagation	Spike-FlowNet, a deep hybrid neural network, integrating SNNs and ANNs for efficiently estimating optical flow from sparse event camera outputs without sacrificing the performance was proposed. They trained the IF neuron with spike-base backpropagation.	On the MVSEC dataset, Spike-FlowNet accurately predicts the optical flow from discrete and asynchronous event streams along with substantial benefits in terms of computational efficiency compared to the corresponding ANN architecture. Code available: https://github.com/chan8972/Spike-FlowNet (accessed on 27 August 2021)
STaRFlow [106] (2021)	Backpropagation	STaRFlow is a lightweight CNN for multi-frame optical flow estimation with occlusion handling. Temporal information is exploited by temporal recurrence, where the same weights over a scale recurrence are repeatedly used.	STaRFlow obtains SOTA performances on MPI Sintel and Kitti2015 and involves significantly fewer parameters. Code available: https://github.com/pgodet/star_flow (accessed on 5 September 2021)

**Table 3:** Summary of recent applications of SNNs in robotics.

Method Year	Software/ Hardware	Description	Performance
Pattern Generation			
Cuevas-Arteaga et al. [107] (2017)	PyNN/SpiNNaker	Spiking CPG (sCPG) to command a hexapod robot to perform walk, trot, or run.	
NeuroPod [81] (2019)	-/SpiNNaker	The first implementation of a real-time neuromorphic sCPG that runs on the SpiNNaker to command a hexapod robot to perform walk, trot, or run.	The robot was based on the design from https://www.thingiverse.com/thing:1021540 (accessed on 5 September 2021)
Strohmer et al. [82] (2020)	NEST/-	An sCPG with AdEx neuron model that exhibits a tripod-like gait. Their model can manipulate the amplitude, frequency, and phase while the network is running, indicating that these characteristics can be updated in an online manner to further explore the role of sensory feedback in shaping locomotion.	A validation test was performed on the Modular Robot Framework (MORF), and source code is available at: https://gitlab.com/esrl/scpg-network-simulation (accessed on 4 September 2021)
Donati et al. [83] (2014)	-/FPGA	Implement an sCPG using analog/digital VLSI device interfaced with an FPGA board, which can be directly interfaced to the actuators of a bio-mimetic robotic lamprey. CPG network is implemented using silicon neurons and synapses with biologically plausible time constants.	The neuromorphic chip can reproduce the behavior of the theoretical CPG model, offering the possibility of directly controling the actuators of an artificial bio-inspired lamprey robot. The neuron produces periodic bursting, lasting approximately 60 ms, with an inter-burst interval of about 1.5 s. The spiking frequency during the burst is about 35 Hz.
Angelidis et al. [84] (2021)	Nengo/SpiNNaker	Used the sCPG model implemented in Nengo to produce the swimming gaits modulated by the high-level brainstem control of a simulated lamprey robot model in various scenarios. They showed that the robot can be controlled dynamically in direction and pace by modifying the input to the network.	The experiments are conducted on an isolated CPG model and neuromechanical simulations. It provides a vast number of possible synchronized gaits, e.g., traveling and standing waves, and smoothly controls a lamprey robot that, with regulation of the high-level drive, adapts to various simulation scenarios. On neuromorphic hardware, it achieves real-time operation.
Motor Control			
Dupeyroux et al. [85] (2020)	PySNN/Loihi	This is a fully embedded application of the Loihi neuromorphic chip prototype in a flying robot. It uses an evolutionary algorithm to optimize SNN controllers and an abstracted simulation environment for evaluation.	The resulting network architecture consists of only 35 neurons distributed among three layers. Quantitative analysis between simulation and Loihi reveals a root-mean-square error of the thrust setpoint as low as 0.005 g, along with a 99.8% matching of the spike sequences in the hidden layer and 99.7% in the output layer. Videos of the flight tests can be found at https://mavlab.tudelft.nl/loihi/ (accessed on 5 September 2021)
Stagsted et al. [86] (2020)	Nengo/Loihi	By modifying SNN architecture and improving the interface between neuromorphic cores and the host computer allowed, it improves the latency and frequency of the controller. The integral path of the controller was re-designed to cope with a limited resolution of value representation. The inner control loop was removed to simplify the network, and the time step duration of the control loop was decreased to improve the I/O interface.	SNN-based proportional, integral, derivative (PID) controller was tested on a drone constrained to rotate on a single axis. They achieved comparable performance for overshoot, rise and settling times.
Navigation			
Spiking RatSLAM [89] (2012)	Spatial Envelope Synthesis (SES)	It demonstrates a model of rat hippocampus place, grid, and border cells implemented with the SpiNNaker. The implemented place cells were used to represent the location of landmarks for “home” and “cheese” whereas the grid cells provide displacement information to the robot. They showed that the robot can detect these landmarks correctly.	Place cells represent the location of landmarks for “home” and “cheese”, while Grid cells provide displacement information to the robot. The experiment shows that that robot is correctly able to detect these landmarks http://neuromorphs.net/nm/wiki/act12/results/Combined (accessed on 5 September 2021)
Gridbot [90] (2018)	ROS/-	Gridbot is an autonomously moving robot with 1321 spiking neurons and is able to map the environment by itself. Gridbot contains neurons that were modeled as LIF units; synapses that were either hardwired or underwent plastic changes through STDP, dendritic trees that integrated synaptic inputs. Gridbot encoded sensory information into distributed maps and generated motor commands to control the robot movement.	Three experiments: follow the walls of the environment for 30 min; explored the environment randomly; the robot walked through the learned environment for more than 2 h
Bing et al. [108] (2019)	-/Kapoho-Bay USB chipset	It is a fast method to build an SNN-based controller for performing robotic implementations by using a model-based control method to shape a desired behavior of the robot as a dataset and then use it to train an SNN based on supervised learning.	It performed slightly better than the state-of-the-art thanks to generalization introduced by Poisson spike encoding of the state input
Tang et al. [88] (2019)	Gazebo/-	The model has two sensory spike rate-encoders and five sub-networks (head direction, reference frame transformation, distance mapping, observation likelihood, Bayesian inference). All five sub-networks are integrated, and the model has intrinsic asynchronous parallelism by incorporating spiking neurons, multi-compartmental dendritic trees, and plastic synapses, all of which are supported by Loihi.	A mobile robot is equipped with an RGB-depth camera, in both the AprilTag real-world and Gazebo simulator, for validating our method. It is validated for accuracy and energy-efficiency in both real- world and simulated environments by comparing with the GMapping algorithm. It consumes 100 times less energy than GMapping run on a CPU while having comparable accuracy in the head direction localization and map-generation.
SDDPG [91] (2020)	PyTroch/-	Spiking deep deterministic policy gradient (SDDPG), which consists of a spiking actor network and a deep critic network that were trained jointly using gradient descent for energy-efficient mapless navigation.	The model was validated with Turtlebot2 platform and Intel’s Kapoho-Bay USB chipset. https://github.com/combra-lab/spiking-ddpg-mapless-navigation (accessed on 6 September 2021)

**Table 4:** List of available software frameworks for SNNs simulation.

Framework	Training Paradigm	Description
SNN simulator		
Brain2 [109]	STDP	Brian2 is a widely-used open-source simulator for spiking neural networks. https://opensourcelibs.com/lib/brian2 (accessed on 6 September 2021)
Nest [110]	STDP/RSTDP	Nest focuses on the dynamics and structure of neural systems, and it is used in medical/biological applications but maps poorly to large datasets and deep learning.
Nengo [111]	STDP PES	Neural simulator for large-scale neural networks based on the neural engineering framework (NEF), which is a large-scale modeling approach that can leverage single neuron models to build neural networks.
Nengo DL [77]	ANN conversion	NengoDL allows users to construct biologically detailed neural models, intermixed with deep-learning elements bucked by TensorFlow and Keras [112].
SpykeTorch [113]	STDP/RSTDP	SpykeTorch is based on PyTorch [114] and simulates convolutional SNNs with at most one spike per neuron and the rank-order encoding scheme.
BindsNet [115]	STDP/RSTDP ANN conversion	BindsNet is also based on PyTorch targeting machine-learning tasks. Currently, synapses are implemented without their own dynamics.
Slayer PyTorch [116]	BP	Slayer PyTorch provides solutions for the temporal credit problem of spiking neurons that allows backpropagation of errors.
Norse	BPTT RSNN	Norse is an expansion of PyTorch to perform deep learning with spiking neural networks using sparse and event-driven hardware and data. Used in long short-term spiking neural networks (Bellec 2018). https://github.com/norse/norse (accessed on 7 September 2021)
snn_toolbox [67]	ANN conversion	SNN toolbox is used to transform rate-based ANNs defined in different deep-learning frameworks, such as TensorFlow, PyTorch, etc., into SNNs and provides an interface to several backends for simulation (pyNN, Brian2, etc.) or deployment (SpiNNaker, Loihi). https://github.com/NeuromorphicProcessorProject/snn_toolbox (accessed on 7 September 2021)
GeNN [117]	SNN	GeNN provides an interface for simulating SNNs on NVIDIA GPUs by generating model-driven and hardware-specific C/C++ CUDA code. https://genn-team.github.io/ (accessed on 7 September 2021)
PySNN	STDP	PySNN focuses on having an efficient simulation of SNN on both CPU and GPU. Written on top of PyTorch to achieve this. https://github.com/BasBuller/PySNN (accessed on 7 September 2021)
CARLsim [118,119]	STP STDP	CARLsim allows th euser to simulate large-scale SNNs using multiple GPUs and CPU cores concurrently. The simulator provides a PyNN-like programming interface in C/C++, which allows for details and parameters to be specified at the synapse, neuron, and network level. https://github.com/UCI-CARL/CARLsim5 (accessed on 4 September 2021)
Auryn [120]	STDP	Auyrn is a simulator for a recurrent spiking neural network with synaptic plasticity. https://github.com/fzenke/auryn (accessed on 5 September 2021)
SNN-based brain simulator		
Neucube [121]	STDP	NeuCube is the development environment and a computational architecture for the creation of brain-like artificial intelligence. https://kedri.aut.ac.nz/R-and-D-Systems/neucube (accessed on 4 September 2021)
FNS [122]	STDP	FNS is an event-driven spiking neural network framework oriented to data-driven brain simulations. https://www.fnsneuralsimulator.org/ (accessed on 6 September 2021)

## Figures and Tables

**Table 1:** A comparison of properties between biological neural networks, ANNs, and SNNs.

Properties	Biological NNs	ANNs	SNNs
Information Representation	Spikes	Scalars	Spikes
Learning Paradigm	Synaptic plasticity	BP	Plasticity/BP
Platform	Brain	VLSI	Neuromorphic VLSI

## Data Availability

Not applicable.
